# *Aspergillus oryzae* and *Aspergillus niger* Co-Cultivation Extract Affects In Vitro Degradation, Fermentation Characteristics, and Bacterial Composition in a Diet-Specific Manner

**DOI:** 10.3390/ani11051248

**Published:** 2021-04-26

**Authors:** Fanlin Kong, Na Lu, Yanfang Liu, Shu Zhang, Hongqin Jiang, Haomin Wang, Wei Wang, Shengli Li

**Affiliations:** 1 Beijing Engineering Technology Research Center of Raw Milk Quality and Safety Control, The State Key Laboratory of Animal Nutrition, College of Animal Science and Technology, China Agricultural University, Beijing 100193, China; fanlinkong1126@163.com (F.K.); Luna2020@cau.edu.cn (N.L.); 13611235024@163.com (Y.L.); zhangshu200666@126.com (S.Z.); 2 China Representative Office, Ascor Chimici S.R.L., 201199 Bologna, Italy; jianghongqin@ascorcn.com (H.J.); wanghaomin@ascorcn.com (H.W.)

**Keywords:** *Aspergillus oryzae*, *Aspergillus niger*, roughage, total mixed ration, digestibility, 16S rRNA

## Abstract

**Simple Summary:**

The rumen is a large fermentation chamber that enables dairy cows to utilize otherwise indigestible plant polymers and compounds for their nutrition, highlighting a crucial difference between ruminants and monogastrics. The key point in this process is the pool of enzymes secreted by microorganisms. Hence, exogenous enzymes from additives are important for high-production dairy cows to improve the utilization of feeds. In this study, we used the *Aspergillus oryzae* and *Aspergillus niger* co-cultivation extract (AOAN) to improve the digestibility of roughage and total mixed ration in vitro. Our results indicated that the digestibility of nutrients in feeds was significantly increased by AOAN supplementation, except for the digestibility of crude protein in the total mixed ratio (TMR). Furthermore, the diversity of the bacteria in TMR and oat hay was increased with AOAN supplementation. Although broad effectiveness of AOAN was established, regardless of roughage types, the mechanism may be different according to roughage types.

**Abstract:**

AOAN may provide enzymes to improve the digestibility of feeds and enhance rumen fermentation. This study determined the effects of AOAN on digestibility, fermentation characteristics, and bacterial composition using in vitro gas recording fermentation system. A total of 30 mg of AOAN was supplemented into 500 mg of TMR, corn silage, oat hay, and alfalfa hay. Fermentation parameters and bacterial communities were determined after 48 h fermentation, and digestibility was determined after 7, 24, 30, and 48 h fermentation. Gas production and dry matter (DM), crude protein (CP), neutral detergent fiber (NDF), and acid detergent fiber (ADF) digestibility were significantly increased by AOAN supplementation at 48 h (*p* < 0.05), except for digestibility of CP of the TMR (*p* > 0.05). AOAN increased starch digestibility in corn silage (*p* < 0.05) and tended to increase that in TMR (0.05 < *p* < 0.10). AOAN supplementation increased total volatile fatty acid production (*p* < 0.05). The molar proportions of acetate and acetate to propionate ratio of oat hay and alfalfa hay were increased (*p* < 0.05). The 16S rRNA analysis revealed that the microbial richness of TMR and oat hay, and microbial evenness of TMR were increased (*p* < 0.05). AOAN did not affect the α diversity, β diversity, and bacterial composition of the corn silage. The relative abundance of *Prevotella* was increased and *Ruminococcus* was decreased in TMR, oat hay, and alfalfa hay. In conclusion, results suggest that AOAN has the potential to improve the utilization of diets differently, including providing enzymes with changing microbiota (TMR, oat hay, and alfalfa hay) or providing enzymes alone (corn silage).

## 1. Introduction

Fibrous roughages account for 40 to 100% of the basic ration of dairy cows. Despite its low energy and nutrient density, there are physiological and economic reasons for increasing the percentage of forage in lactating cow’s diet. However, if higher forage-based diets are to be realized, then increasing the rate and extent of fiber digestion is critical to meet the energetic requirements of copious milk production. Diet fungal products can enhance rumen fermentation and alter ruminal digestive processes; thus, they may improve animal health and energy status in early lactation [1]. *Aspergillus oryzae* and *Aspergillus niger* fungi have been chosen for production in food science in a low-cost medium [2,3,4,5] and both have a long history concerning strain improvement to optimize enzyme production [6,7,8,9]. Thus, *A. oryzae* and *A. niger* co-cultivation extract (AOAN) has potential as a feed additive, but their use has long been ignored in dairy feeding.

Several studies have investigated the efficacy of *A. oryzae* or *A. niger* on improving the nutritional quality of grape seed [10], palm kernel cake [11], ginkgo leaves [12], and common feeds [13,14] through fermentation. However, inconsistent results have been obtained from animal experiments when fed *A. oryzae* or *A. niger* extract alone [15,16,17]. These results may be due to different diet types, which may provide limited substrate for enzymes secreted from *A. oryzae* or *A. niger* to degrade. The effects of *A. oryzae* on rumen fermentation and microbial populations have been proven to be determined by the roughage type [13]. In addition, the single enzyme secreted by only one fungus may also contribute to inefficient results from animal experiments. The different strains enhance the nutritional composition in varying degrees [10]. Digestion of plants occurs extracellularly for fungi. Hence, it is important for the natural degradation of plant biomass by enzymes produced by several *Aspergilli* spp. Hu et al. [18] reported that co-cultivation of *A. oryzae* and *A. niger* resulted in improved production of β-glucosidase, cellobiohydrolase, β-xylosidase, and laccase. However, studies are lacking on the effects of AOAN on nutrient digestibility, fermentation characteristics, and bacterial composition using the 16s rRNA sequencing approach, which is widely used in dairy nutrition to characterize prokaryotic communities [19,20,21].

A common issue with all in vitro experiments is the paucity of in vivo data. However, it must be noted that in vitro techniques can provide satisfactory accuracy for comparative purposes and precludes unnecessary in vivo experimentation [22,23]. Therefore, we used an in vitro experiment to investigate the effects of supplementation of AOAN on nutrient degradation of a total mixed ration (TMR), three different roughages (corn silage, oat hay, and alfalfa hay), at four-time points (7 h, 24 h, 30 h, and 48 h). The fermentation characteristics, gas production, and bacterial composition at 48 h were also determined. We hypothesized that AOAN could affect the microbiome, and subsequently affect dietary degradation. The current study will provide fundamental information about AOAN and investigate the potential of AOAN as a feed additive, which to date has been ignored in the dairy industry.

## 2. Materials and Methods

### 2.1. Animals

Three multiparous, rumen-cannulated, lactating, Holstein dairy cows (550 ± 25.4 kg body weight; 67 ± 9 days in milk; 32 ± 2.67 kg/d milk yield; mean ± standard deviation) were used to provide rumen fluid at China Zhongdi Dairy Holdings Company Limited in Beijing. The main ingredients and chemical composition of the TMR are shown in Table 1. The cows were fed three times daily at 0730, 1330, and 1830, and milked three times daily at 0900, 1500, and 2000. All animal procedures were approved by the Institutional Animal Care and Use Committee of the China Agricultural University (CAU20201009-2).

### 2.2. Experiment Design and In Vitro Batch Culture

The in vitro experiments in anaerobic glass bottles (volume capacity of 120 mL) combined with the Automated Trace Gas Recording System for Microbial Fermentation (AGRS III, Beijing, China), incubated with a substrate, buffer solution, and rumen fluid were performed according to the description of Zhang and Yang [24]. The fermentation system in each bottle included 500 mg substrate, 50 mL rumen fluid, and 25 mL buffer solution. The rumen fluid from each cow was collected 2 h after morning feeding, filtered through four layers of cheesecloth, transported in a pre-warmed thermos bottle at 39 °C, and then mixed in the laboratory with CO_2_ at 39 °C. The rumen fluid sampling process was conducted according to Yáñez-Ruiz et al. [25]. The buffer solution was prepared following the method of Menke [26]. After mixing, the buffer solution was bubbled with CO_2_ until the pH value was 6.8.

The substrates consisted of a TMR, corn silage, oat hay, and alfalfa hay obtained from the same commercial farm where the rumen-cannulated dairy cows resided. The fresh TMR sample was collected after mixing (MVS-16-H self-propelled vertical double auger, TATOMA, Monzón, Spain). The ingredients of TMR are shown in Table 1. The corn silage, oat hay, and alfalfa hay samples were taken from the silage pit or feed warehouse. All the samples were taken from five points and mixed. After 2 h transportation from the farm to the laboratory, TMR and corn silage were dried at 60 °C for 48 h, and then all feeds were ground to pass through a 2 mm screen (FNF-400, Qingdao Jimo, and Pulverizer Plant, Shandong, China). The chemical compositions are shown in Table 2.

The treatments included control (no AOAN treatment) and 60 mg/g AOAN. The fermentation substrates were TMR, corn silage, oat hay, and alfalfa hay, respectively. Finally, according to the substrate amount (500 mg each bottle), 30 mg AOAN was weighted into each bottle. The AOAN product was supplied by Bioscreen Technologies SRL (Bologna, Italy). The additive amount of AOAN was referred to Sun et al. [13]. The main ingredient of the commercial product was corncob power with *A. oryzae* and *A. niger* fermentation (94.5% dry matter, 26% crude protein, 4.2% crude fiber, 3.8% crude fat). The TMR and corn silage fermentation included four-time points (7 h, 24 h, 30 h, 48 h), and hay or alfalfa hay fermentation included three-time points (24 h, 30 h, 48 h). Each time point included six replicates. These bottles were immediately connected to the Automated Trace Gas Recording System. Three bottles without substrate were used as the blank.

### 2.3. Sample Collection and Measurement

After 7 h, 24 h, 30 h, and 48 h of incubation, the content of each sample was filtered through a nylon bag (80 mm × 150 mm size with 42 μm pores), and then dried at 60 °C for 48 h in a forced-air oven (Wujiang Zhongda Electrical Technology Co., Ltd., Wujiang, Jiangsu, China) to analyze and calculate nutrient degradability. The content of dry matter (DM), neutral detergent fiber (NDF), acid detergent fiber (ADF), and crude protein (CP) in the original sample, and residues at 24 h, 30 h, and 48 h were determined according to the previously described method [27]. The starch content of residues of TMR and corn silage at 7 h was determined using a commercial starch assay kit (BioVision, Inc., San Francisco Bay Area, the United States of America). Starch is hydrolyzed to glucose, which is oxidized to color at 570 nm and can be read by a microplate reader (Multiskan Sky Microplate spectrophotometer, Thermo Fisher Scientific-CN, Shanghai, China).

The culture fluid at 48 h was sampled from four of the six samples in each group into 2.5 mL microtubes and stored in liquid N for DNA extraction. The 15 mL fluid subsample was stored at −80 °C for ammonia nitrogen (NH_3_-N) and volatile fatty acid (VFA) analysis [28]. Briefly, the culture fluid at 48 h for VFA was centrifuged at 4731× *g* (5400 rpm, 14.5 cm) for 10 min, and the supernatant (1 mL) mixed with 200 μL metaphosphoric acid solution (25%, *w*/*v*). After shaking in an ice-water bath for 30 min, samples were centrifuged at 5595× *g* (10,000 rpm, 5 cm) for 10 min. The supernatant was injected into gas chromatography (Agilent 6890N, Agilent Technologies, Inc., Beijing, China) to determine the concentrations of acetate, propionate, butyrate, and branched fatty acids. The content of individual VFAs is shown as a molar proportion. The subsamples for NH_3_-N analysis were centrifuged at 2000× *g* for 20 min at 4 °C, and the supernatant (2 mL) was acidified with 8 mL of 0.2 N hydrochloric acid. The pH at 48 h was detected immediately using a portable pH meter (S2-Meter, Mettler Toledo International Co., Ltd., Shanghai, Beijing, China). The experimental design is shown in Supplemental Figure S1.

### 2.4. DNA Extraction and Sequencing

Bacterial DNA was extracted from 48 h samples using an Omega Stool DNA kit (Omega Bio-Tek, Norcross, GA, USA). The quality and quantity of the DNA were determined using NanoDrop 2000 UV–vis spectrophotometer (Thermo Scientific, Wilmington, DE, USA). Amplicon library preparation was performed by polymerase chain reaction (PCR) of the V3-V4 region of the 16s rRNA gene using universal primers 338F (5′-ACTCCTACGGGAGGCAGCAG-3′), and reverse primers 306R (5′-GGACTACHVGGGTWTCTAAT-3′) [28]. The 20 μL reaction system included 10 ng of template DNA, 4 μL of FasPfu buffer, 2 μL of 2.5 mmol/L dNTPs, 0.8 μL of each primer, 0.4 μL of FasPfu polymerase, 0.2 μL bovine serum albumin, and double-distilled H_2_O to 20 μL. The amplicons were electrophoresed on 2% agarose gel, purified using an Agencourt AM Pure XP kit (Beckman Coulter Genomics, Indianapolis, IN, USA), and quantified using the Quanti-FluorTM-ST system (Promega, Madison, WI, USA). Finally, the purified amplicons were pooled in equimolar concentrations and pair-end sequenced on an Illumina MiSeq platform (Illumina, Inc., San Diego, CA, USA).

### 2.5. Sequencing Data Processing

The raw sequencing data were filtered and processed using the Quantitative Insights into Ecology (QIIME) program (1.9.0) [29]. The sequences were classified into operational taxonomic units (OTUs) following the threshold of 97% identity using USTRA-fast sequence analysis (version 10.0.240) [30], and the OTU numbers were assigned based on unique OTU reads. Taxonomy classifications were assigned against the Silva bacterial alignment database [31], with a confidence threshold of 70%, using the Ribosomal database project classifier [32].

Alpha diversity indices were calculated to indicate community diversity through QIIME [29]. Differences in Chao1, Ace, numbers of OTUs, and Shannon indices were analyzed with the Mann–Whitney U test and the *p*-value was calculated, corrected for the false discovery rate, on the Microbiome Analyst platform [33]. The principal coordinates analysis (PCoA) was conducted based on the Bray–Curtis distance on the Microbiome Analyst platform [33]. Analysis of non-parametric multivariate of variance (PERMANOVA) was calculated using the Bray-Curtis distance metric (permutation = 999). The linear discriminant analysis effect size (LEfSe) in the Microbiome Analyst platform was used to identify genera that showed significant differences in relative abundance [34]. The raw reads were deposited at NCBI (under BioProject accession ID: PRJNA699978, RUN: SRR13696322-SRR13696353, https://www.ncbi.nlm.nih.gov/Traces/study/?acc=PRJNA699978, (accessed on 12 February 2021)

### 2.6. Statistical Analyses

The gas production data (gas production, mL/g, dry matter basis) exported from the automated recording system in Excel were fitted according to an exponential model as described by France et al. [35].

(1) $${\mathrm{GP}=A\;\times \;[1\;-\;e}^{-C\;\times \;\left(\mathrm{time}\;-\;\mathrm{Lag}\right)}]$$

where GP (mL) is the gas production, A (mL) is the ideal maximum gas production, C (h^−1^) is the gas production rate, Lag (mL) is the lag phase before gas production commences. The nonlinear regression procedure in statistical analysis system (SAS) 9.2 (SAS Institute Inc., Cary, NC, USA) was used in this process. The time taken to reach half of the ideal maximum gas production (HT, h), and average gas production rate when half of the ideal maximum gas production produced (AGPR, mL/h) were calculated as shown below according to the A, C, and Lag values obtained above [36]:

(2) $$\mathrm{HT}=\log\left(\frac{2}{C}\right)+\mathrm{Lag}$$

(3) $$\mathrm{AGPR}=\frac{A\;\times \;C}{2\;\times (\log\left(2\right)+C\;\times \mathrm{Lag})}$$

The data, including starch digestibility, fermentation parameters, and gas production kinetics parameters, were analyzed using the general linear model produce (GLM) of SAS 9.2 to obtain the effects of feed type, AOAN, and feed type × AOAN. The DM, CP, NDF, and ADF digestibility at 24 h, 30 h, and 48 h were analyzed using GLM produce as described above to investigate the effects of feed type, AOAN, time, and AOAN × time. All data are presented as least-squares means. All differences were declared significant at *p* ≤ 0.05, and tendencies at 0.05 ≤ *p* ≤ 0.10.

To assess the correlation between the phenotypic variables and the relative abundance of microbial genera, the Spearman correlation test was performed using SPSS 20.0 (IBM, Armonk, NY, USA), and plotted using GraphPad Prism 7 (GraphPad Software, San Diego, CA, USA). For each correlation, the correlation coefficient value ranged from −1 to +1 with larger absolute values indicating a stronger relationship and positive/negative values indicating the direction of the association.

## 3. Results

### 3.1. Gas Production Kinetics Parameters

**Figure 1:**
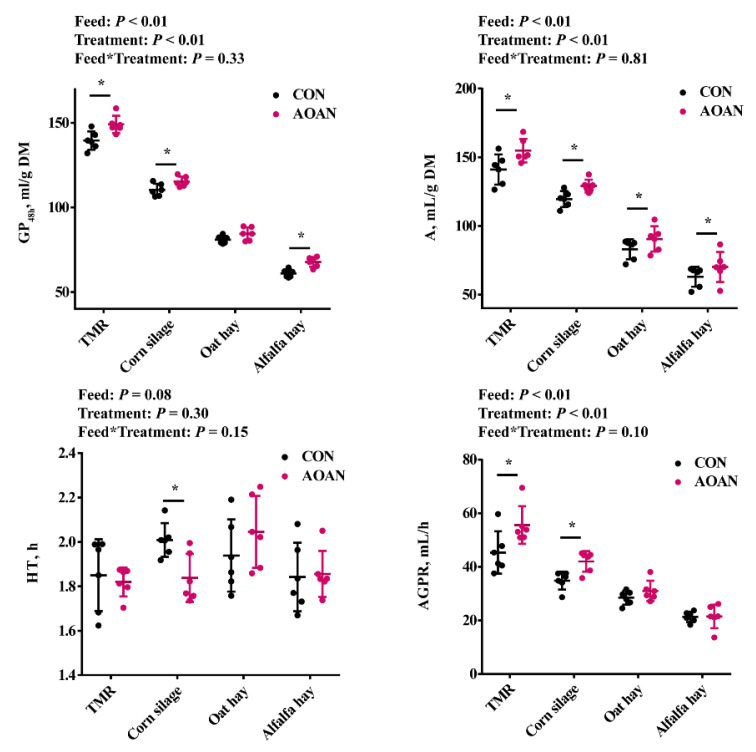
Effects of *Aspergillus oryzae* and *Aspergillus niger* co-cultivation fermentation extract on in vitro gas production kinetics parameters of a total mixed ration, corn silage, oat hay, and alfalfa hay. GP_48h_, cumulative gas production at 48 h; A, ideal maximum gas production; HT, Time to reach half the ideal maximum gas production; AGPR, average gas production rate when half of the ideal maximum gas production was produced; CON, feedstuff fermentation with no additive using the in vitro gas production technique; AOAN, feedstuff fermentation with *Aspergillus oryzae* and *Aspergillus niger* co-cultivation fermentation extract using the in vitro gas production technique; *, *p* < 0.05; TMR, total mixed ration.

The effects of AOAN supplementation on the gas production kinetics parameters of the TMR and the three different roughages are shown in Figure 1. The gas production and A at 48 h in the TMR, corn silage, and alfalfa hay were significantly increased by AOAN supplementation (*p* < 0.05). The A of oat hay was increased by AOAN supplementation (*p* < 0.05). Interestingly, the AGPR of TMR and corn silage increased by AOAN supplementation (*p* < 0.05).

### 3.2. Nutrient Digestibility

**Figure 2:**
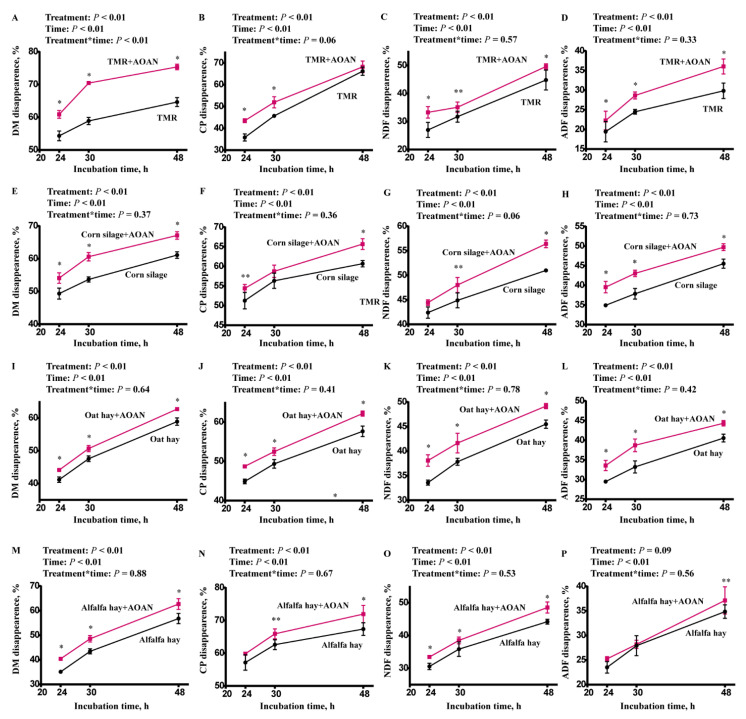
Effects of *Aspergillus oryzae* and *Aspergillus niger* co-cultivation fermentation extract on in vitro nutrient disappearance of total mixed ration (**A**–**D**), corn silage (**E**–**H**), oat hay (**I**–**L**), and alfalfa hay (**M**–**P**). AOAN indicates fermentation with *Aspergillus oryzae* and *Aspergillus niger* co-cultivation fermentation extract. *, *p* < 0.05; **, 0.05 < *p* < 0.1; TMR, total mixed ratio; DM, dry matter; CP, crude protein; NDF, neutral detergent fiber; ADF, acid detergent fiber.

Figure 2 shows the effects of AOAN supplementation on DM, CP, NDF, and ADF digestibility at 48 h. Nutrient digestibility was higher when AOAN was supplied at three of the time points. All DM digestibility was increased by AOAN supplementation (*p* < 0.05). At 48 h, the CP digestibility of corn silage, oat hay, and alfalfa hay was increased by AOAN supplementation (*p* < 0.05). The NDF and ADF digestibility at 48 h of all feeds increased (*p* < 0.05), except for ADF digestibility of alfalfa hay (0.05 < *p* < 0.10). All variables were affected by time (*p* < 0.05). However, no interaction between AOAN supplementation and time was observed in most variables (*p* > 0.05), except for the interaction in DM digestibility of TMR (*p* < 0.05).

**Figure 3:**
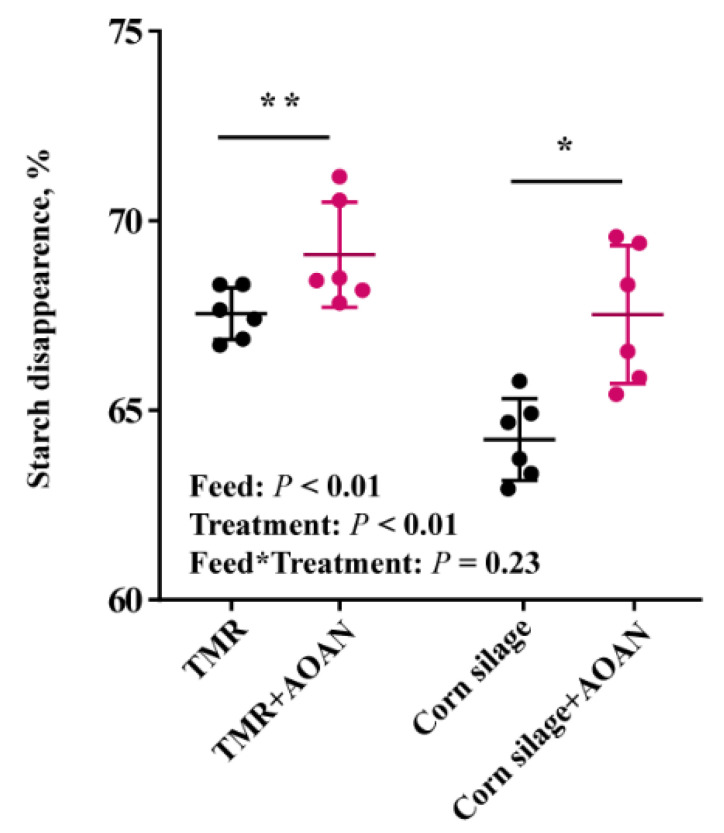
Effects of *Aspergillus oryzae* and *Aspergillus niger* co-cultivation fermentation extract on starch disappearance in total mixed ration and corn silage at 7 h in vitro incubation. AOAN indicates fermentation with *Aspergillus oryzae* and *Aspergillus niger* co-cultivation fermentation extract. *, *p* < 0.05; **, 0.05 < *p* < 0.1; TMR, total mixed ration.

The starch digestibility at 7 h in the TMR and corn silage was analyzed (Figure 3). There was a difference in corn silage starch digestibility (*p* < 0.05) and a trend in the TMR (0.05 < *p* < 0.10). None of them had an interaction between feed and AOAN supplementation (*p* > 0.05).

### 3.3. Fermentation Parameters

**Figure 4:**
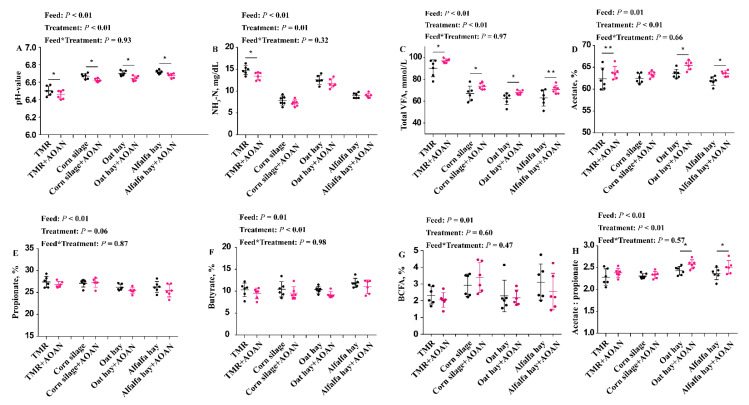
Effects of *Aspergillus oryzae* and *Aspergillus niger* co-cultivation fermentation extract on in vitro fermentation parameters ((**A**): pH value; (**B**): NH_3_-N; (**C**): total volatile fatty acid; (**D**–**G**): individual VFA; (**H**): acetate: propionate) of total mixed ration, corn silage, oat hay, and alfalfa hay. AOAN indicates fermentation with *Aspergillus oryzae* and *Aspergillus niger* co-cultivation fermentation extract and individual VFAs are represented as molar proportions. *, *p* < 0.05; **, 0.05 < *p* < 0.10; TMR, total mixed ration; NH_3_-N, ammonia nitrogen; VFA, volatile fatty acid; BCFA, branched chain fatty acids (isobutyric + isovaleric).

Figure 4 shows the effects of AOAN supplementation on fermentation parameters. The pH values were decreased (Figure 4A; *p* < 0.05), and NH_3_-N concentration in TMR was decreased with AOAN (Figure 4B; *p* < 0.05). The total VFAs were increased or tented to be increased by AOAN (Figure 4C). There were no differences in the molar proportions of propionate and butyrate (Figure 4E,F; *p* > 0.05). AOAN increased the molar proportion of acetate, and the acetate to propionate ratio of oat hay and alfalfa hay (Figure 4D,H; *p* < 0.05). The molar proportion of total branched fatty acid including isobutyric and isovaleric was not affected by treatment (Figure 4G; *p* > 0.05).

### 3.4. Bacterial Community

#### 3.4.1. Sequence Depth, Diversity, and Composition

After data filtering, a total of 2,263,946 reads from 32 samples were obtained with a mean of 70,748 reads for individual samples. The Good’s coverage for each sample was deemed sufficient, with values > 99.00% for all bacterial communities, implying that the current sequencing depth was sufficient to be representative of the microbiota (Supplemental Figure S2. The sample-based rarefaction curves in Supplemental Figure S3 indicate that our sequencing depth was sufficient to accurately describe the bacterial composition of all groups.

**Figure 5:**
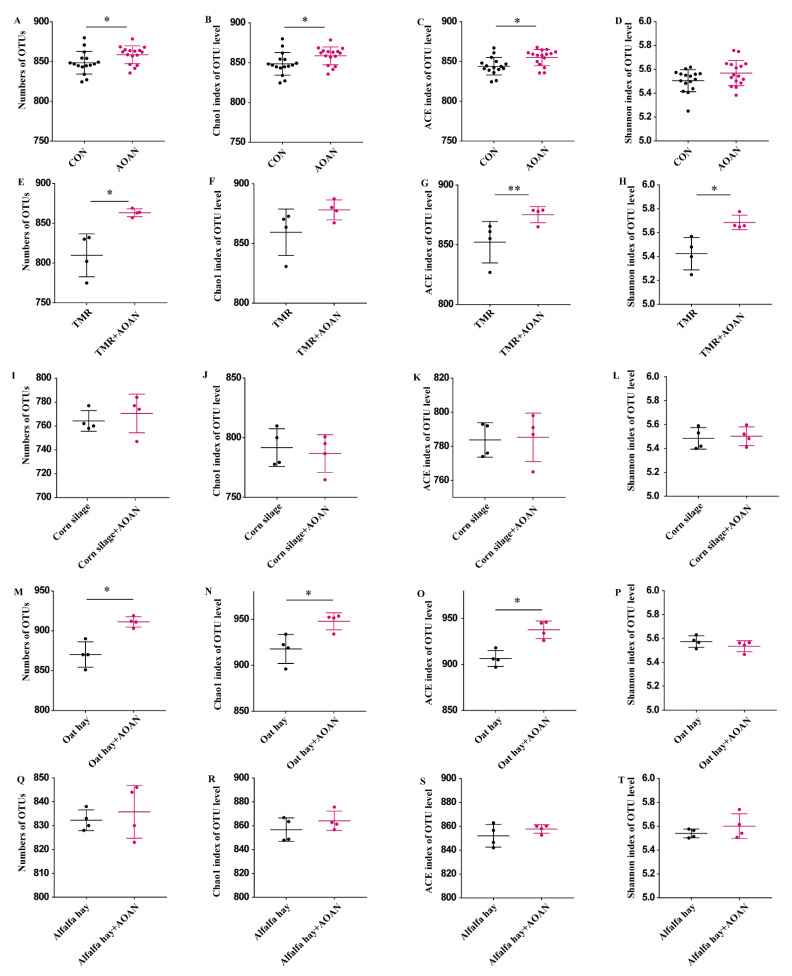
Effects of *Aspergillus oryzae* and *Aspergillus niger* co-cultivation fermentation extract on α diversity based on OTU level of TMR (**E**–**H**), corn silage (**I**–**L**), oat hay (**M**–**P**), alfalfa hay (**Q**–**T**), and all (**A**–**D**). AOAN indicates fermentation with *Aspergillus oryzae* and *Aspergillus niger* co-cultivation fermentation extract. CON group indicates all feeds without AOAN supplementation. AOAN group indicates all feeds with AOAN supplementation. *, *p* < 0.05; **, 0.05 < *p* < 0.1. TMR, total mixed ration.

The α diversity based on OTU level is shown in Figure 5 as the CON group (all feeds without AOAN supplementation) vs. AOAN group (all feeds with AOAN supplementation) (Figure 5A–D) or individual feed vs. AOAN + individual feed (TMR, Figure 5E–H; corn silage, Figure 5I–L; oat hay, Figure 5M–P; alfalfa hay, Figure 5Q–T). The OTU number, Chao1, and ACE of CON and oat hay were increased by AOAN supplementation (*p* < 0.05). The OTU number and Shannon of TMR were increased by AOAN supplementation (*p* < 0.05). The α diversity of corn silage and alfalfa hay was not affected by treatment (*p* > 0.05).

#### 3.4.2. The β Diversity and Bacterial Composition

**Figure 6:**
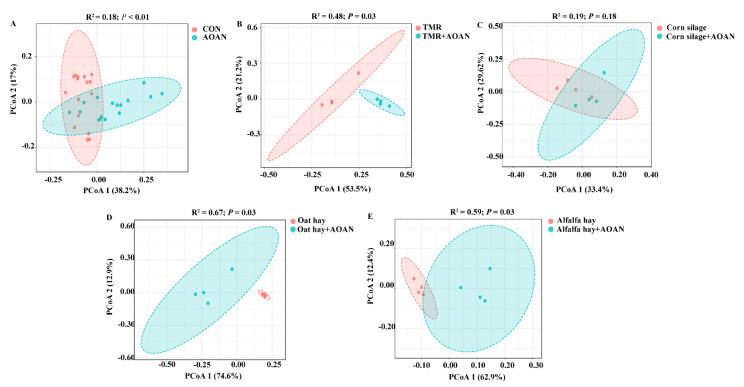
Effects of *Aspergillus oryzae* and *Aspergillus niger* co-cultivation fermentation extract on β diversity calculated using Bray–Curtis dissimilarity, based on OTU level of TMR (**B**), corn silage (**C**), oat hay (**D**), alfalfa hay (**E**), and all (**A**). Groups were significantly different using PERMANOVA analysis. AOAN indicates fermentation with *Aspergillus oryzae* and *Aspergillus niger* co-cultivation fermentation extract. CON group indicates all feeds without AOAN supplementation. AOAN group indicates all feeds with AOAN supplementation. TMR, total mixed ration.

Figure 6 shows β diversity based on OTU level. The PERMANOVA showed that the bacteria were different, except in corn silage (Figure 6A,B,D,E; *p* < 0.05). The PCoA showed visual separations in Figure 6A,B,D,E, except for corn silage (Figure 6C).

**Figure 7:**
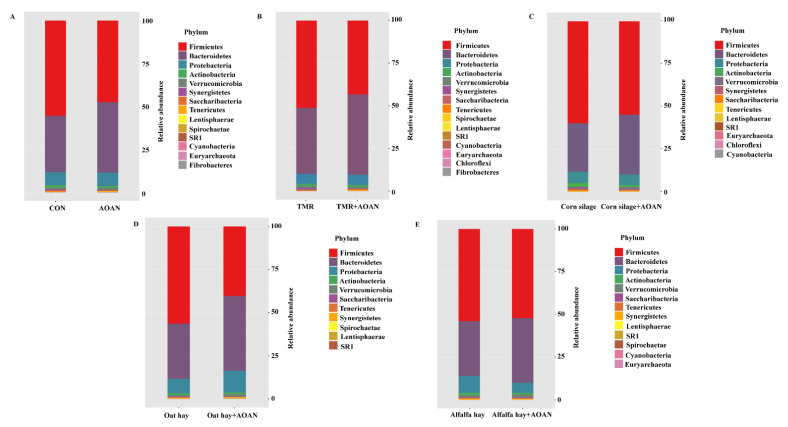
Effects of *Aspergillus oryzae* and *Aspergillus niger* co-cultivation fermentation extract on the composition of phyla of bacterial communities in a TMR (**B**), corn silage (**C**), oat hay (**D**), alfalfa hay (**E**), and all (**A**). AOAN indicates fermentation with *Aspergillus oryzae* and *Aspergillus niger* co-cultivation fermentation extract. CON group indicates all feeds without AOAN supplementation. AOAN group indicates all feeds with AOAN supplementation. TMR, total mixed ration.

The dominant bacterial phyla (relative abundance > 1%) included Firmicutes (51.76 ± 7.51%), Bacteroidetes (36.67 ± 7.10%), Proteobacteria (7.54 ± 2.79%), and Actinobacteria (1.30 ± 0.52%) (Figure 7).

#### 3.4.3. Differential Genera

**Figure 8:**
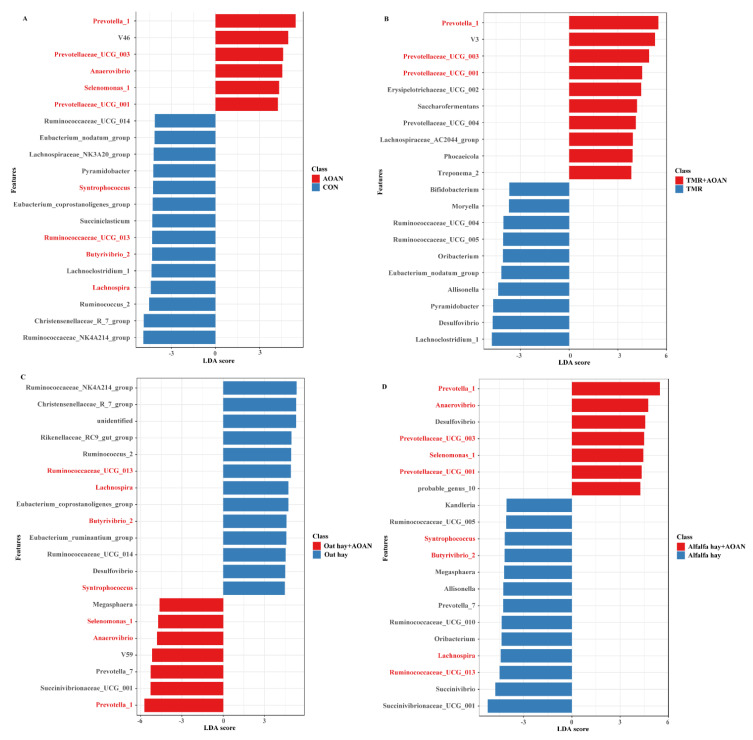
Differential bacterial genera between CON (all feeds without AOAN supplementation) and AOAN (all feeds with AOAN supplementation) (**A**), TMR and TMR + AOAN (**B**), oat hay and oat hay + AOAN (**C**), alfalfa hay and alfalfa hay + AOAN (**D**). AOAN indicated feed fermentation with *Aspergillus oryzae* and *Aspergillus niger* co-cultivation fermentation extract. CON group indicates all feeds without AOAN supplementation. AOAN group indicates all feeds with AOAN supplementation. Significant differences were tested by linear discriminant analysis effect size (LEfSe) and declared as |linear discriminant analysis score| > 2 and adjusted-*p* < 0.10. The genera in red indicate that the genus was enriched more than three times. TMR, total mixed ration.

At the genus level, the top 20 differential genera between two different groups were examined by |LDA| score > 2 and adjusted-*p* < 0.10 (Figure 8). There were no significant differences in the corn silage group with AOAN supplementation (*p* > 0.10, |LDA| score < 2). *Prevotella_1* belonging to Bacteroidetes exhibited higher relative abundance following AOAN supplementation (Figure 8A–D; *p* < 0.10). The genera *Prevotellaceae_UCG-003* and *Prevotellaceae_UCG-001* belonging to Bacteroidetes in CON, TMR, and alfalfa hay (Figure 8A,B,D), and *Selenomonas_1* and *Anaerovibrio* belonging to Firmicutes in CON, oat hay, and alfalfa hay (Figure 8A,C,D) were increased (*p* < 0.10). The genera *Syntrophococcus*, *Ruminococcaceae_UCG-013*, *Lachnospira*, and *Butyrivibrio_2* belonging to Firmicutes in CON, oat hay, and alfalfa hay were decreased by AOAN supplementation (Figure 8A,C,D; *p* < 0.10).

#### 3.4.4. Spearman Correlation Analysis

**Figure 9:**
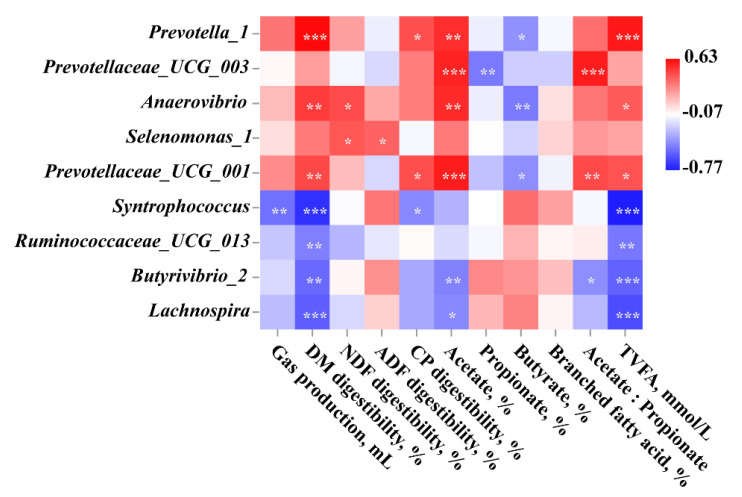
Spearman correlation between the relative abundance of genus features and phenotypic indices. Genus features obtained using Linear Discriminant Analysis Effect Size analysis from AOAN vs. CON. AOAN indicated feed fermentation with *Aspergillus oryzae* and *Aspergillus niger* co-cultivation fermentation extract. CON group indicates all feeds without AOAN supplementation. AOAN group indicates all feeds with AOAN supplementation. The color represents the correlation coefficient. Red represents a positive correlation, and blue represents a negative correlation. A dark color represents a stronger correlation, and a light color represents a weaker correlation. *, 0.01 < *p* < 0.05; **, 0.001 < *p* < 0.01; ***, *p* ≤ 0.001; DM, dry matter; CP, crude protein; NDF, neutral detergent fiber; ADF, acid detergent fiber; TVFA, total volatile fatty acid.

Spearman correlation analysis between the relative abundance of genus features from AOAN vs. CON and phenotypic indices is shown in Figure 9. The results show that the relative abundance of *Prevotella_1*, *Prevotella_UCG_001*, and *Anaerovibrio* were positively correlated with DM digestibility, TVFA, and molar proportion of acetate (*p* < 0.05), and negatively correlated with the molar proportion of butyrate (*p* < 0.05). The relative abundance of *Prevotella_UCG_003* and *Prevotella_UCG_001* were positively correlated with acetate to propionate ratio (*p* < 0.05). There was a significant negative correlation between DM digestibility, TVFA, and the relative abundance of *Syntrophococcus*, *Ruminococcaceae_UCG_013*, *Butyrivibrio_2*, and *Lachnospira* (*p* < 0.05).

## 4. Discussion

Our objective was to evaluate the effects of adding AOAN on nutrient digestibility of a TMR and three roughages (corn silage, oat hay, and alfalfa hay) typical of those given to dairy cows in China by analyzing fermentation parameters and bacterial composition (Supplemental Figure S1).

NDF digestibility after 30 h of incubation and starch digestibility after 7 h of incubation were related to the value and quality of feed [37,38]. AOAN significantly improved nutrient utilization and digestion. Several studies have examined the *A. oryzae* product, Amaferm, and have indicated that Amaferm increased NDF digestibility of switchgrass and bromegrass at 12 h [39], alfalfa hay at 48 h [40], and DM digestibility of a TMR at 24 h in vitro [41]. Positive results from the animal experiment also indicated that DM and CP digestibility were increased by Amaferm supplementation [42], and fiber digestibility of the rumen and total tract were improved [43]. Our results are consistent with these studies. However, the inconsistent results from Sievert et al. [17], who indicated that total tract digestibility was not changed and milk fat was depressed by Amaferm. Lactation cows are different from dry cows, which might partly explain the divergent results. Furthermore, a recent study reported that the percentage and types of roughage used could influence this response [13]. Many reasons may contribute to this response, including different strains used in fermentation or unique chemical linkages in the roughage [10,44]. Therefore, we used an extract from *A. oryzae* combined with *A. niger* to improve the use of fungal extracts. Although the results obtained by in vitro techniques cannot completely reflect or replace those obtained from in vivo studies. The results of the present study provide important insights into the efficacy of AOAN.

GP and rate are indicators of fermentability and digestibility. Khazaal et al. [45] reported that GP increased with in vitro digestibility. In our study, the GP and ideal maximum GP were also increased with nutrient digestibility. The single most striking observation was that the HT of TMR was minimum and AGPR was maximum in these feeds, which may be attributed to the balance between energy generation from fermentable carbohydrates and nitrogen generation from fermentable nitrogen in the TMR. Overall, these results show that AOAN was effective to improve the nutritional value of different feeds and was characterized by its broad-spectrum.

The acetate in the rumen, fat precursors, is associated with milk fat production. In our study, the higher molar proportion of acetate and higher acetate to propionate ratio were observed in oat hay and alfalfa hay by adding AOAN, and then this change may contribute to milk fat production by supplying acetate. In animal experiments, the milk fat depression was obtained from Sievert and Shaver [17] by *A. oryzae* extract, and Zicarelli et al. [46] by *Saccharomyces cerevisiae* plus *A. oryzae* extract. According to Campanile et al. [47], milk fat depression could be due to the increased organic matter digestibility, which allowed higher energy availability for milk yield and reduced the fat mobilization during the first phase of lactation. The propionate in the rumen, glucose precursors, is associated with energy supply for maintenance requirement and milking. In our study, the higher starch digestibility and unchanged acetate to propionate ratio of corn silage remind us that acetate and propionate might be increased at the same time. Hence, we suggest that the AOAN not only increases acetate for milk fat production but also increases propionate for energy. However, Higginbotham et al. [48] found no significant differences in milk yield in dairy cows fed a diet supplemented with *A. oryzae* extract. Thus, an animal experiment is essential to confirm these reflections of milk performance in the future.

The AOAN or other commercial fungal extracts contain inactivated microorganisms, their products, and the medium. The comprehensive enzymes in their products could stimulate the growth of microorganisms by converting macromolecular nutrients to available nutrients for microorganisms, which increase the nutrient digestibility of the substrate and produce a large quantity of VFA to decrease pH in the medium [39,49]. Alternatively, the inactivated microorganisms and medium also provide limited nutrients for microorganisms and then enhance the nutrient digestibility, as evidenced in other fungi [50]. Regardless of the pathway, the intention is to improve the diversity and function of microorganisms through AOAN supplementation. In the current study, AOAN improved the bacterial diversity in total, which was consistent with our digestibility data. Further analysis indicated that the differences were mainly derived from TMR and oat hay rather than corn silage and alfalfa hay. In addition, the increment was mainly driven by an increment in richness (number of OTUs, Chao1, and ACE), rather than evenness. In general, oat hay has the same contents of NDF and CP as corn silage, whereas corn silage contains a higher level of starch as readily fermentable carbohydrates, which are fermented within 7 h and long before 48 h [38]. Further work on bacterial composition at 7 h would be meaningful to investigate the effects of AOAN on corn silage fermentation. In summary, these results suggest that AOAN is effective in improving bacterial diversity and is more significant for TMR and oat hay.

The increasing digestibility of feeds suggested that more substrate was provided for bacteria in this study. Many other studies have reported that the NH_3_-N concentration is not changed by *A. oryzae* extract [40,43,49,51,52]. The NH_3_-N concentration depends on the balance between generation and consumption. The significant increase in bacterial diversity in the TMR indicates that bacterial communities may grow and utilize NH_3_-N to produce microprotein, which may partly explain the decreased NH_3_-N concentration in TMR, and further suggests that AOAN may be given to modify microorganisms more effectively than *A. oryzae* extract alone. However, the rumen undegraded protein in the feed may be decreased and the effects of AOAN on metabolizable protein including rumen undegraded protein and microprotein is still not clear. Zicarelli et al. [46] observed no differences in terms of milk yield using *A. oryzae* (20 g/head/d) but a lower percentage of protein content in milk, while the milk protein yield was not calculated according to the numerical increase of milk production. Hence, further investigation is needed to investigate the effects of AOAN supplementation on the metabolizable protein of feedstuff with different protein quality.

Similar to previous studies using 16S rRNA sequencing in vitro, the most abundant bacteria at the phylum level that were shared across all culture solutions were Firmicutes, Bacteroidetes, and Proteobacteria [53,54]. Previously, an in vitro experiment using bromegrass and switchgrass fermented with *A. oryzae* extract reported that cellulolytic organisms were 3.5 times higher than that of the control [39]. The dairy cows fed *A. oryzae* extract also had higher counts of cellulolytic bacteria and proteolytic bacteria [55]. With the development of bacterial culture technology, Beharka and Nagaraja [56] provided the effects in detail and found *A. oryzae* extract increased the growth rate of cellulolytic bacteria (*Ruminococcus albus* and *Fibrobacter succinogenes*), and lactate-utilizing bacteria (*Megasphaera elsdenii*, *Selenomonas lactilytica*, and *Selenomonas ruminantium*), which were consistent with the results from Sun et al. [13,15], and the increased relative abundance of *Selenomonas_1* in our study. However, those studies used classical, culture-based, microbiology methods, which describe only a small fraction of the total bacterial population [57]. In our study, the results were obtained from *Prevotella*, which would previously have been described as both cellulolytic bacteria and proteolytic bacteria because at least a third of glycoside, hydrolases, and other carbohydrate enzymes are affiliated with *Prevotella* [58]. This genus utilizes starch, protein, and fiber to produce succinate and acetate, and is one of the most abundant core genera in the rumen of dairy cows. Thus, it was not surprising to identify the positive correlation between the relative abundance of *Prevotella* and molar proportion of acetate, and *Prevotella* might play a key role in DM digestibility and TVFA production. In addition, *Anaerovibrio* has commonly been reported to be involved in lipolysis, and the lipase from *A. niger* extract may release fatty acids in the substrate and contribute to the increased relative abundance [59,60]. The positive correlation between the relative abundance of *Anaerovibrio* and the molar proportion of acetate implied that lipolysis in this bacteria could produce acetate.

Following the addition of AOAN, a significant decrease in the relative abundance of *Ruminococcus* and the negative correlation between this genus and DM digestibility were not expected, in disagreement with previous studies using *A. oryzae* extract [13,15,56]. The abundance of *Ruminococcus albus* that increased in previous studies is just one of the species in this genus. Furthermore, other results showed that the counts of microorganisms may increase with total VFA production and increased α diversity. Therefore, the actual amount of *Ruminococcus* rather than relative abundance may increase. Furthermore, the fermentation system is a complex ecosystem composed of anaerobic, bacterial, fungi, protozoa, methanogenic archaea, and phages. Other microorganisms besides bacteria may influence the relative abundance of *Ruminococcus*.

Like *Ruminococcus*, most of the decreased genera, including *Syntrophococcus*, *Desulfovibrio*, and *Lachnospira* were carbohydrate-degrading bacteria and acetogenic bacteria. *Butyrivibrio_2* is responsible for producing butyrate from glucose fermentation or acetate. The negative correlation between relative abundance of *Butyrivibrio_2* and molar proportion of acetate suggested that this genus might be considered as acetate-utilization bacteria. However, a pure culture study indicated that *Prevotella* grew more abundantly in the presence of water-soluble cellulose acetate, yielding enhanced levels of acetate [61]. These increased *Prevotella* spp. might be the main contributors to the higher molar proportion of acetate. In summary, our sequencing results indicate that AOAN is beneficial to bacterial diversity and manipulated acetogenic bacteria of TMR, oat hay, and alfalfa hay fermentation rather than corn silage to change the fermentation pattern, and *Prevotella* is more sensitive to AOAN.

## 5. Conclusions

In this experiment, the fermentation system used TMR and three different roughages with AOAN supplementation resulted in enhanced bacterial diversity, and further improved nutrient digestibility and VFA production. *Prevotella_1* was enriched in the bacterial community following AOAN supplementation. The results suggest that AOAN is effective in improving the digestibility of feeds. Although the efficacy of AOAN was significant, the unchanged microbiome in corn silage fermentation and enhanced microbial diversity of oat hay fermentation led to the differentiation of the AOAN pathway. An in vivo study in the future is needed to confirm our results, to determine the appropriate inclusion in diet, and extend the use of AOAN.

## Figures and Tables

**Table 1:** Ingredients of total mixed ration used in vitro (dry matter basis).

Ingredient	Level (%)
Corn	4.95
Soybean hull	2.78
Soybean meal	7.61
Molasses beet	2.23
Whole cottonseed	2.88
NaHCO_3_	0.28
Yeast powder	0.06
Steam flaked corn	9.74
Alfalfa hay	7.93
Whole corn silage	50.07
Rumen-pass fatty acid	0.42
Corn bran	2.70
Distillers dried grains and soluble	4.17
Oat hay	2.31
Mycotoxin remover agent	0.06
Premix ^1^	1.81

^1^ One kg premix contained the following: VA, 130,000 IU; VE, 465 IU; Cu, 2600 mg; Mn, 6000 mg; Zn, 2600 mg; Se, 70 mg; I, 120 mg; Co, 70 mg.

**Table 2:** Chemical composition of total mixed ration and feedstuffs used in vitro (dry matter basis).

Item ^1^	TMR	Corn Silage	Oat Hay	Alfalfa Hay
DM, %	94.6 ± 0.3	93.5 ± 0.2	94.1 ± 0.2	93.4 ± 0.3
CP, %	16.0 ± 0.3	8.6 ± 0.1	6.2 ± 0.2	21.4 ± 0.5
NDF, %	38.2 ± 0.6	54.0 ± 0.9	56.9 ± 1.1	41.1 ± 0.6
ADF, %	25.3 ± 0.4	34.3 ± 0.6	33.9 ± 0.4	26.6 ± 0.3
Starch, %	26.8 ± 0.3	28.6 ± 0.2	-	-

^1^ DM: dry matter; CP: crude protein; NDF: neutral detergent fiber; ADF: acid detergent fiber. Chemical composition was obtained from chemical analysis and shown as mean ± standard deviation. ‘-’ indicates no data.

## Data Availability

The data presented in this study is available on request from the corresponding author. The raw reads were deposited at NCBI (under BioProject accession ID: PRJNA699978, RUN: SRR13696322-SRR13696353).

## Supplementary Materials

The following are available online at https://www.mdpi.com/article/10.3390/ani11051248/s1. Figure S1: Experimental design and workflow, Figure S2: The good coverage for each sample, Figure S3: The samples rarefaction curve based on observed species.
