# Supplementary material for: Aspergillus oryzae and Aspergillus niger Co-Cultivation Extract Affects In Vitro Degradation, Fermentation Characteristics, and Bacterial Composition in a Diet-Specific Manner
Source: Animals (Basel). 2021 Apr 26;11(5):1248. doi: 10.3390/ani11051248 (PMC8145302; doi:10.3390/ani11051248)
Supplement: Supplementary file 1 [file animals-11-01248-s001.zip › animals-1167397-SI-final.pdf]

animals

MDPI

Article

Aspergillus oryzae and *Aspergillus niger* co-cultivation extract affects *in vitro* degradation, fermentation characteristics, and bacterial composition in a diet-specific manner

Fanlin Kong [1,†], Na Lu [1,†], Yanfang Liu [1], Shu Zhang [1], Hongqin Jiang [2], Haomin Wang [2], Wei Wang [1,*] and Shengli Li [1,*]

[1] The state Key Laboratory of Animal Nutrition, Beijing Engineering Technology Research Center of Raw Milk Quality and Safety Control, Collage of Animal Science and Technology, China agricultural University, Beijing, 100193, China; fanlinkong1126@163.com (F.K.); Luna2020@cau.edu.cn (N.L.); 13611235024@163.com (Y.L.); zhangshu200666@126.com (S.Z.).

[2] China Representative Office, Ascor Chimici S.R.L. Bologna, 201199, Italy, jianghongqin@ascorcn.com (H.J.); wanghaomin@ascorcn.com (H.M.).

[*] Correspondence: lisheng0677@163.com, Tel: +86-(10)-62731254 (S.L.); wei.wang@cau.edu.cn (W.W.), Tel.: +86-(10)-18610013227.

[†] The author contributed equally to this research.

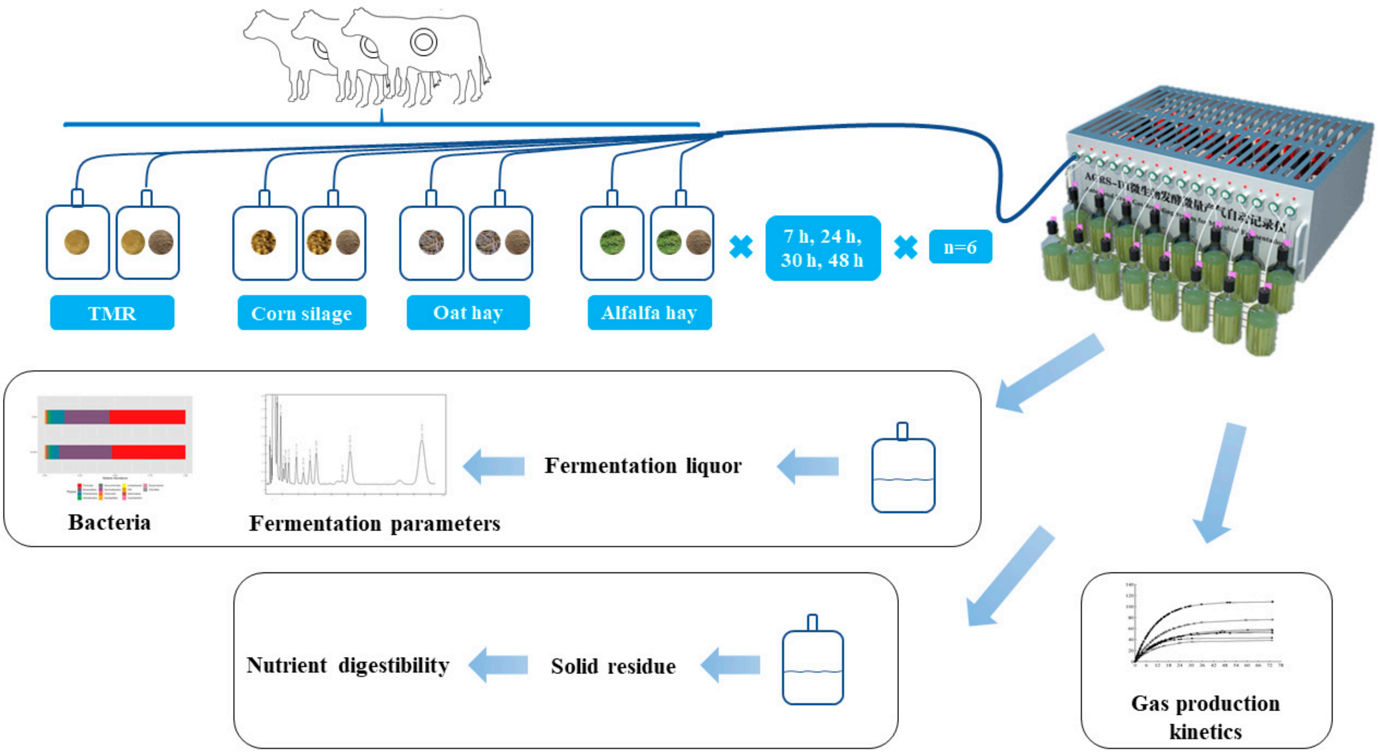

Figure S1. Experimental design and workflow.

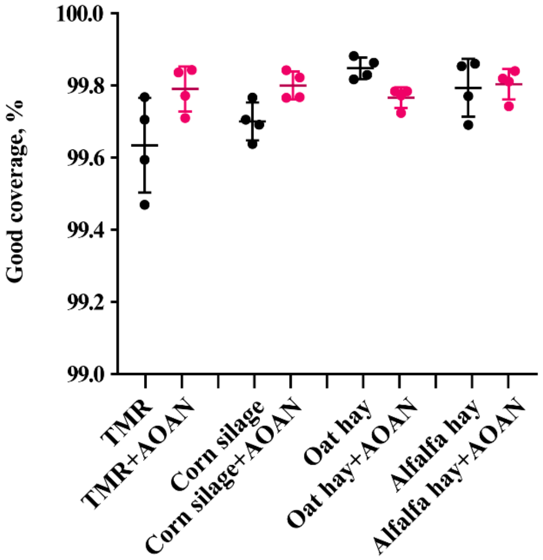

Figure S2. The good coverage for each sample. AOAN indicated feed fermentation with Aspergillus oryzae and Aspergillus niger fermentation extract. TMR, total mixed ration.

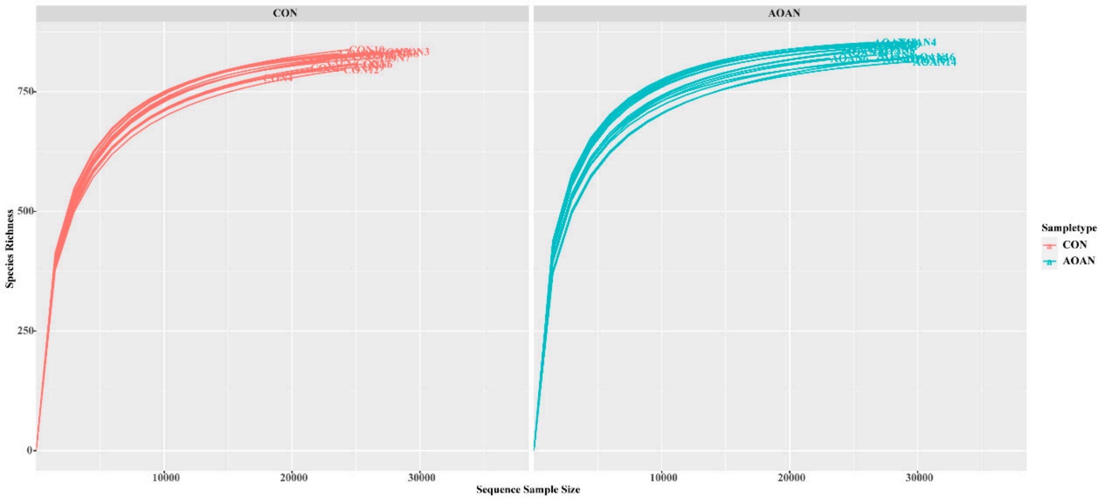

Figure S3. The samples rarefaction curve based on observed species. CON indicates all feeds fermented without AOAN supplementation and AOAN indicates all feeds fermented with AOAN supplementation. AOAN indicated feed fermentation with Aspergillus oryzae and Aspergillus niger fermentation extract.
